# Effects of exercise training on bone mineral density and some health-related outcomes in HIV conditions: A randomized controlled trial

**DOI:** 10.1097/MD.0000000000023206

**Published:** 2020-12-11

**Authors:** Adedayo Tunde Ajidahun, Hellen Myezwa, Sam Chidi Ibeneme, Sebastian Magobotha, Gerhard Fortwengel, Maxwell Jingo, Brenda Milner, Sadiya Ravat, Ifeoma Okoye, Edward Schnaid, Faith Bischoff

**Affiliations:** a Department of Physiotherapy, University of the Witwatersrand, Johannesburg, South Africa; b Department of Medical Rehabilitation, Faculty of Health Sciences and Technology, College of Medicine, University of Nigeria, Enugu Campus. Nigeria; c Department of Orthopaedic Surgery, University of the Witwatersrand, Johannesburg, South Africa; d Hochschule Hannover – University of Applied Sciences and Arts, Hannover, Germany; e University of Nigeria Teaching Hospital Enugu (UNTH), Ituku-Ozalla, Enugu State, Nigeria.

**Keywords:** HIV, bone mineral density, fracture and osteoporosis

## Abstract

**Introduction:**

Human Immunodeficiency Virus (HIV) infection remains prevalent co-morbidity, and among fracture patients. Few studies have investigated the role of exercise interventions in preventing bone demineralization in people who have fractures and HIV. If exercise exposed, HIV-infected individuals may experience improved bone health outcomes (BMD), function, quality of life (QoL). The study will aim to assess the impact of home based exercises on bone mineral density, functional capacity, QoL, and some serological markers of health in HIV infection among Nigerians and South Africans.

**Methods and design:**

The study is an assessor-blinded randomized controlled trial. Patients managed with internal and external fixation for femoral shaft fracture at the study sites will be recruited to participate in the study. The participants will be recruited 2 weeks post-discharge at the follow-up clinic with the orthopaedic surgeon. The study population will consist of all persons with femoral fracture and HIV-positive and negative (HIV-positive medically confirmed) aged 18 to 60 years attending the above-named health facilities. For the HIV-positive participants, a documented positive HIV result, as well as a history of being followed-up at the HIV treatment and care center. A developed home based exercise programme will be implemented in the experimental group while the control group continues with the usual rehabilitation programme. The primary outcome measures will be function, gait, bone mineral density, physical activity, and QoL.

**Discussion:**

The proposed trial will compare the effect of a home-based physical exercise-training programme in the management of femoral fracture to the usual physiotherapy management programmes with specific outcomes of bone mineral density, function, and inflammatory markers.

**Trial registration:**

The study was prospectively registered with the Pan African Clinical Trials Registry (Reference number – PACTR201910562118957) on October 21, 2019. (https://pactr.samrc.ac.za/TrialDisplay.aspx?TrialID=9425).

## 1 Introduction

South Africa, with an estimated population of 56.5 million in 2017^[1]^ is the epicenter of the HIV epidemic in the world.^[2]^ According to the UNAIDS ^[3]^ report, South Africa has an estimated 7.5 million people living with HIV, with 200,000 new infections and 72,000 HIV-related deaths. Currently, South Africa has the most extensive antiretroviral treatment (ART) programme globally, with over 3 million HIV-infected persons.^[2]^ HIV programmes in SA are estimated to cost $1.34 billion annually.^[4]^ Similar to South Africa, Nigeria, with an estimated population of 161.7 million by 2015,^[5]^ has about 3.2 million people living with HIV.^[4]^ Currently, Nigeria records about 180, 000 HIV-related deaths, 227, 000 new HIV cases with the highest incidence occurring in the productive age group (15–49 years).^[4]^ In Nigeria, 51% of adults with HIV are placed on antiretroviral therapy (ART).^[6]^ Highly active antiretroviral therapy (HAART) has significantly increased life expectancy.^[7,8]^ With increased longevity and side effects of HAART, the prevalence of chronic and non-communicable diseases among people living with HIV as part of the ageing process, has been evident.^[9]^

Reduced bone mineral density is a common associated with HIV, and their relationship is multi-factorial. The major related pathogenesis is that HIV infection alters bone metabolism, and this is further compounded with long-term use of HAART.^[10–12]^ HIV was shown to increase the risk of osteoporosis by 3.7, and the long-term use of HAART further increases the risk by 1.6 when the prevalence of osteoporosis was compared between HIV-positive and HIV-negative individuals.^[13]^ Although, the loss of bone mineral density associated with the use of HAART usually stabilizes after one year, and there is no further HAART related bone loss.^[14]^

Apart from HIV and several other risk factors for reduced bone mineral density, immobilization as a result of fracture is also a predisposing factor. A study by Ceroni et al,^[15]^ showed that there is a significant reduction in bone mineral density in patients immobilized using cast compared to healthy controls. The difference in bone mineral density ranged from −5.8% to −31.7% at the hip, greater trochanter, calcaneus, and total lower limb. Also, the consequent immobilization of the affected individuals should translate to loss of independence in activities of daily living, limited functional capacity, loss of economic independence and poor quality of life (QoL).^[16]^ These challenges could minimize functional mobility with an adverse impact on socialization resulting in restricted social participation. The net effect from a population-based national outlook suggests a substantial loss of productive workforce, decline in the overall national productivity, economic development, gross domestic product, and QoL with a predictable negative impact on poverty level.^[17,18]^ Therefore, any interventions that could prevent/ameliorate or reverse the adverse effects of HIV and HAART-related bone demineralization should be of socio-economic significance.

Physical exercise can be defined as (a subset of physical activity) planned, structured and repetitive bodily movement, performed with the goal of improving or maintaining physical fitness.^[19,20]^ Not only does exercise improve health, it also increases muscle strength, coordination, balance, flexibility, and leads to overall health, which may enhance functional capacity in activities of daily living and thus, QoL. Available evidence suggests that exercise is a safe and relevant therapy in HIV/AIDS management, but its safety and relevance have not been fully investigated or exploited to the benefit of the patients.^[21–23]^ The decision on whether to use physical exercises to address the issues related to bone health in HIV conditions should be guided by objective clinical markers. In recent years, biochemical markers (Bone-specific alkaline phosphatase and pyridinoline) have enabled the detection of changes of bone turnover in response to biological (e.g., hormonal), biomechanical, and other variables.^[24,25]^ This trend is useful and may guide therapeutic/clinical decisions for the benefit of clients.

**Figure 1:**
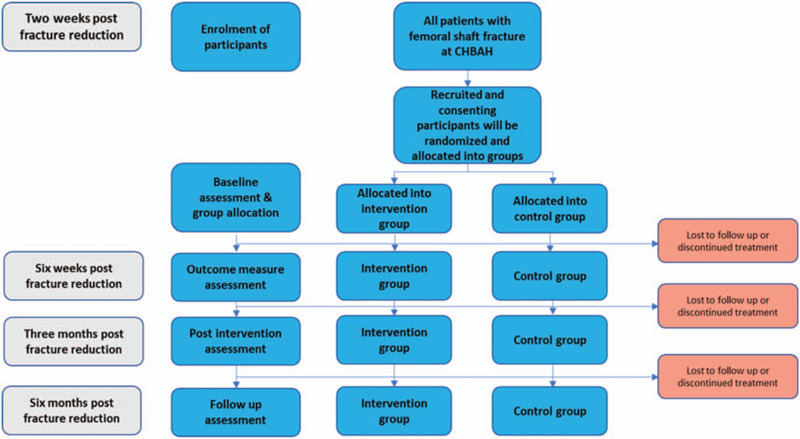
Modified anticipated CONSORT flow diagram for a single-blind randomised controlled trial of physical exercise intervention for bone health in the patients.

We previously described a home-based rehabilitation (HBR) programme we developed in KwaZulu-Natal, South Africa, which adhered to the fundamental principles of a theoretical model of integrated care, that is, the Chetty model, and is also home-based.^[26]^ The Chetty model promotes greater integration and collaboration between hospitals and communities. The HBR programme in KwaZulu-Natal was able to apply several principles such as evidence-based practice, task shifting to lay personnel, enabling patient-centered care and maximising function and independence of People Living with HIV/AIDS (PLWHA). Other elements, such as the adoption of a multidisciplinary approach, training on the use of disability screening tools and the use of evidence to influence policy development were more challenging to implement. However, our findings suggest that it is possible to implement elements of the integrated model of care. The lessons we learned from our KwaZulu-Natal study,^[26]^ and the Nigeria-HIV BMD study,^[27]^ have been explored to determine how the home-based exercise programmes can be deployed to address bone health issues and other contextual disabilities arising from HIV that may be amenable to physical exercises. Also, the small sample studies already cited have demonstrated efficacy and relevance of physical exercises in ameliorating/preventing HIV/HAART-related bone demineralization, while boosting immune function, and serves as an orientation towards a population-based study.^[27–29]^ We will also exploit the opportunity to explore various coping mechanisms of people living with HIV using an adaptation of the COPE questionnaire, which may have implications for QoL.^[30]^ The objective of this article is to describe the protocol of a study, which is aimed at determining the effects of exercise on bone mineral density and other health outcomes. The proposed study design is randomized controlled trial, as outlined in Figure 1.

## 2 Aim and objectives of the study

The study will aim to determine the effectiveness of a home-based exercise programme in the management of femoral fracture. The specific objectives of the study will be to:

1. To develop an evidence-based exercise intervention programme, based on evidence from the literature for improved: BMD, immune and inflammatory markers, BMI, function capacity and HRQOL
2. To determine the effects of physical exercises on BMD, immune and inflammatory markers and functional capacity in adults living with HIV in Nigeria & South Africa with femoral shaft fracture.

## 3 Hypothesis

It is hypothesized that:

1. Home based exercise programme will significantly influence muscle strength, gait output/speed, BMD, functional capacity, body composition (%BFM, FM, FFM, and BMI) and immune function (CD^4+^ count) in HAART-experienced HIV-infected adults compared to controls in both Nigeria and South Africa.
2. HAART may have a significant interaction effect with physical activity to improve BMD in HAART-experienced HIV-infected adults compared to controls in Nigeria and South Africa.

## 4 Research significance

The proposed trial will compare the effect of a home-based physical exercise-training programme in the management of femoral fracture to the usual physiotherapy management programmes with specific outcomes of BMD function and inflammatory markers.

## 5 Methods

### 5.1 Phase one

The first phase of the study will aim to develop an appropriate home-based exercise intervention programme based on evidence from the literature for improved: BMD, immune and inflammatory markers, BMI, function capacity, and HRQOL.

*Study design and procedure*: A comprehensive literature review will be performed to extract evidence on existing exercise-based intervention programme that has been used in the management of lower limb fractures. The concept of the home-based intervention programme will focus on the following—the joint range of motion, muscle strengthening, weight-bearing, proprioception, and gait training. After the exercise programmes have been developed, the programme will be validated with physiotherapists with a speciality in orthopaedics and neuro-musculoskeletal systems. The home-based programme will include cognitive and behavioral strategies that will consist of a discussion of benefits and barriers to exercise and individualized goal setting and self-monitoring of progress by using the exercise adherence and compliance booklet.

### 5.2 Phase two

The second phase of the study will aim to determine the effects of physical exercises on BMD, immune and inflammatory markers and functional capacity in adults living with HIV in Nigeria and South Africa with femoral shaft fracture.

*Trial design:* The proposed study design is a multi-center parallel group assessor-blinded randomized controlled trial with allocation ratio of 1:1.

### 5.3 Study setting

*Site 1*: The study will be conducted at Chris Hani Baragwanath Academic Hospital (CHBAH), Johannesburg, South Africa, which is a multidisciplinary teaching hospital for the University of the Witwatersrand, Faculty of Health Sciences. Gauteng, with an estimated population of 14.3 million, is one of the nine provinces of South Africa, with Johannesburg as its biggest city with 4.4 million inhabitants.^[1]^

*Site 2:* This is primarily at the Enugu State University Teaching Hospital, Parklane and the University of Nigeria Teaching Hospital, Enugu State, Nigeria. Enugu State, with an estimated population of 3.8 million, is one of the 36 states of Nigeria. The state has an HIV prevalence of 1.3% (Male: 1.0%; Female: 1.6%).^[31]^ There are 20,297 people living with HIV on treatment in 21 treatment facilities.^[31]^ HIV services are offered in 318 centers.^[31]^ The state benefits from the expanded access to ART instituted in Nigeria in 1999 from a national coverage for 359,181 people in 2010 to 747,382 in 2014.^[32]^ Since then, the prevalence of HIV/AIDS in Enugu has risen from 1.3% in 1992, to 5.2% in 2001, and 6.5% in 2005, but declined remarkably to 1.3% in 2012 suggesting that HIV transmission has not shown a stable pattern of decline over the past decades.^[31,33]^

### 5.4 Eligibility criteria

The study population will consist of all persons with femoral fracture and HIV-positive and negative (HIV-positive medically confirmed) aged 18 to 60 years attending the above-named health facilities. For the HIV-positive participants, a documented positive HIV result, as well as a history of being followed-up at the HIV treatment and care center.

Inclusion criteria

- Only patients between 18 and 60 years at the last birthday and not more than 60 years at the next birthday.
- For HIV-negative participants, willingness to accept HIV testing.
- All patients on ART therapy with femoral shaft fracture will be recruited.
- All patients with lower limb fractures (femur) will be recruited.
- All patients must be ambulant.

Exclusion criteria

- Severely ill persons based on investigator judgment.
- Presence of any bone or rheumatic disorder.
- Fracture of the femoral neck.

*Interventions*: The intervention programme will be a home-based exercise programme targeted at improving bone mineral density, function, gait, QoL, and physical activity. The intervention programme aims to improve the joint range of motion, gait training, muscle strengthening, and balance. The participants in the intervention group will be followed up weekly via phone calls to monitor the intervention and ensure adherence to the programme.

*Control group*: The control group will continue with their usual rehabilitation programme as conducted by the various clinics.

### 5.5 Outcomes

Table 1 outlines the outcome measure, tools, validity, and reliability for the study.

**Table 1:** Psychometric measures.

Outcome measure	Instrument	Reliability and validity
Bone mineral density	Portable Xrite 331C densitometer (quantitative ultrasound densitometer)	The Portable Xrite 331C densitometer is valid instrument to measure bone density. The test-retest reliability of the portable densitometer will be determined in this study.
Laboratory tests	**Bone resorption**: Serum Beta-CTx or Carboxy-terminal telopeptide cross-linked type 1 collagen (CTX) **Bone formation**: osteocalcin **Pro-inflammatory markers**: C-reactive protein (CRP) **HIV clinical markers:** HIV test, CD4 count, viral load and albumin **Other bone metabolism biomarkers**: calcium and magnesium	The test will be performed using the Roche Cobas 8000 analyser. The tests have high repeatability using human samples in accordance to the Clinical and Laboratory Standards Institute (CLSI) EP5 requirements.
Functional capacity	WHODAS^[34]^	WHODAS is a valid and reliable (α = 0.94) tool used in measuring physical and functional health^[34]^
Health-related quality of life	EQ-5D questionnaire^[35]^	EQ-5DL is a valid and reliable tool (ICC = 0.87) in chronic musculoskeletal problems.^[36,37,38]^
Gait speed	4 m gait speed	The 4 m gait speed test is a reliable (ICC = 0.715) and valid test for measuring general health and muscle mass ^[39]^.
Gross muscle strength	Hand held isokinetic dynamometer *r* = 0.40 – 0.71; intertester ICC = 0.82– 0.84	The handheld dynamometer is a valid and reliable tool in assessing gross muscle strength.^[40–42]^ Intertester and intratester reliability—ICC = 0.82 – 0.8 and ICC = 0.91 – 0.94. The HHD shows a significant positive correlation with step length and reaction time (*r* = 0.40 – 0.71; *P* < .05) ^[41]^.
Anthropometric measures (weight, height and BMI)	Stadiometer and weighing scale	The stadiometer and weighing scale will be calibrated before each use.
Body composition	Fat loss monitor with scale (OMRON HBF – 400)	Weight is measured in 0.2 lb increments. Body fat percentage is measured from 5% to 60% in 0.1% increments. BMI is rated from 7.0 to 90.0 in 0.1 increments^[43]^.
Physical activity	Short-form International Physical Activity questionnaire (IPAQ-SF) The Yamax Digiwalker SW200 pedometer will be used to objectively quantify physical activity.	The IPAQ-SF is a seven-item valid and reliable tool in measuring self-reported physical activity in the last seven days^[44,45]^. The Yamax Digiwalker SW200 has been used in several epidemiological studies involving the evaluation of free-living physical activity^[46,47]^. The pedometer is considered a reliable and validated tool for intervention and observational studies of physical activity levels among young people^[48]^.
Adherence	Exercise Adherence Rating Scale^[49]^	The exercise adherence rating scale is a valid and reliable (α = 0.81) tool in measuring exercise adherence^[49,50]^. Test-retest reliability, ICC = 0.97^[49]^.
Coping	COPE Inventory^[30]^	The COPE inventory is a reliable and valid tool in assessing coping strategies with chronic problems^[51]^.

### 5.6 Participant

*Sampling and sample size:* Consecutive sampling will be used to recruit the participants. Using the statistical superiority sample size design,^[34]^ a sample of size of 36 participants per group will be sufficient to show a statistical difference in the bone mineral density between the control and the experimental group. For this study, the groups are divided into four which are the experimental group (HIV+ and HIV−) and the control group (HIV+ and HIV−), therefore, at least a total of 144 patients per study site will be recruited to participate in the study. The sample size will be calculated using Equation 1:

(1) $$N=2\times \left\{Z_{1-\alpha /2}+Z_{1-\beta }/d2\right\}\times Pooled\;SD$$

where N = total sample size per group, *Z*_1−α/2_ = 1.96, *Z*_1−β_ = 0.845, *d*^2^ = 0.1 and pooled standard deviation = 0.093.

### 5.7 Recruitment

Patients managed with internal and external fixation for femoral shaft fracture at the study sites will be recruited to participate in the study. The participants will be recruited 2 weeks post-discharge at the follow-up clinic with the orthopaedic surgeon. Consenting participants will be assessed for eligibility and eligible participants will be allocated into the intervention or control group.

### 5.8 Allocation

Blocked allocation will be used to allocate the participants into groups. Random numbers will be generated to allocate the patients into intervention and control groups in blocks (10 numbers equally distributed into intervention and control groups). There will be two blocks namely

1. HIV-positive and
2. HIV-negative blocks.

The number allocation will be done by independent research support. Opaque envelopes will be used to conceal the allocation.

### 5.9 Blinding

The assessors and the data analysts will be blinded to the participant's assigned groups.

### 5.10 Assessments

Baseline and follow up assessment will be conducted by a qualified physiotherapist (Research assistant) who will be blinded to the study.

### 5.11 Procedure

#### 5.11.1 Pilot study

A pilot randomized controlled trials will be conducted to evaluate the feasibility of the study procedure and prepare for unforeseen logistical circumstances. The recruitment process, randomization, intervention protocol, and assessment using the outcome measures exactly as proposed will be tested. A sample of eight participants per group, which is ∼10% of the estimated sample size will be recruited for the pilot trial. After recruitment, baseline (2-weeks post-surgery) assessment of the variables will be measured, followed by a 6-week (post-surgery). The control group will continue with their usual treatment programme. Participants in the experimental and control groups will be re-assessed at the end of the 4-week intervention programme. If there were no changes to the study procedure, the pilot participants would be included in the main study. If there are amendments to the protocol, the changes will be communicated to the relevant bodies.

### 5.12 Main study

The research will recruit patients with femoral fracture being managed at the orthopaedic department at CHBAH, and the following process will be used as a guide for the recruitment, blinding, and allocation. The main study procedure will follow the recruitment, allocation, blinding and intervention guidelines as presented. The CONSORT diagram below in Figure 1 will be used to report the recruitment and allocation of the participants. The time of recruitment and assessment is the usual routine clinic check-up of the patients in the orthopaedic surgery department (2-weeks, 6-weeks, 12-weeks, and 6-months post-surgery).

### 5.13 Data management

All physical measurements, as well as clinical and laboratory data, are will be entered into a case record form (CRF) that is developed prior to the commencement of the study. Instructions on how to complete the relevant forms and how to carry out the various physical, laboratory and radiological measurements are to be provided in appropriate standard operating procedures. The questionnaires and the CRFs will be checked for completeness.

Subjects’ data will be stored on a server located in Berlin, Germany. There will be ongoing data cleaning and verification. The computerized handling of the data by the study team conducting the research after receipt of the data may generate additional requests to which the study sites are obliged to respond by confirming.

The original hard copies of the questionnaires, CRFs, and related documents will be stored securely (at the University of Nigeria and at Chris Hani Baragwanath Academic Hospital, Johannesburg, for Nigeria and South Africa study sites, respectively) in lockable filing cabinets with restricted access, both during and after the completion of the study. The entire study documentation will be stored for a minimum of 5 years after the publication of the primary data.

### 5.14 Reporting serious adverse events

Reports of health deterioration warranting hospitalization or resulting in the death of any participant will be reported to the PI within 24 h. All participants must be informed of such developments within 72 h and be required to re-consent to continue with the trials.

### 5.15 Statistical analysis

Data to be collected shall include discrete and normally distributed continuous variables. The normal distribution of the continuous variables will be tested using the Shapiro–Wilk test, and depending on the outcome, parametric or non-parametric statistics will be used to analyze the data. The change in the categorical outcomes across the time points will be measured using the McNemar's test. The difference in the continuous outcomes between the groups will be measured using the independent *T*-test (parametric) and Mann–Whitney *U* test (non-parametric). Intention to treat analysis method will be used for this study. Alpha shall be set at *P* < .05 (two-tailed). All analyses will be intent-to-treat and missing data will be treated as missing.

## 6 Discussion

Physical exercise could influence bone metabolism and bone healing,^[52,53]^ as demonstrated in studies involving non-HIV infected individuals. There are possible translational benefits for people living with HIV. Exercise interventions may improve function, livelihood and QoL, as well as contribute to reducing hospital costs, enhancing return to work and local and national productivity. Available evidence suggests that co-morbidities associated with degeneration in HIV conditions are worsened by a lack of physical exercise and restriction in social participation leading to multi-system (neurological, musculoskeletal, cardiopulmonary, and metabolic) impairments, which are amenable to physical exercise modalities. The impact of the home-based exercise programme on bone mineral density may reduce the risk of osteoporosis. Previous studies have demonstrated this prospect but are yet to be explored in a large randomized controlled trial. As it is, most of the studies on bone disease and physical exercises in HIV condition have been done outside Africa, which by far has the highest prevalence of HIV infection globally. There are contextual, racial, and gender differences that may influence the outcomes of an exercise intervention on BMD. Such differences can alter the interventions needed, including exercise. For this reason, it is prudent to research to test the impact of an exercise intervention on BMD in HIV positive patients in an African context.

### 6.1 Impact of results

The benefits of exercise interventions in HIV and HAART-related bone demineralization should be explored in a randomized controlled trial. Moreover, limitations in availability, affordability and accessibility of most HIV patients to clinics where gymnasiums are available, may limit compliance to the use and prescription of relevant exercise modalities. Therefore, we consider implementing evidence-based functional-based oriented, simplified, cost-free and innovative home-based exercises and which need to be tested for efficacy in achieving the desired therapeutic goals of improved physical functioning and BMD in HIV management. A home-based rehabilitation approach under an integrated healthcare model, which we developed in a semi-rural poor resource setting of KwaZulu–Natal, was therefore proposed as the most viable option. South Africa and Nigeria are multi-cultural societies and appear as ideal settings for such studies to enable easy recruitment of participants who are exposed to similar environmental variables that may influence bone metabolism other than genetic differences. The net effect of delayed lower limb fracture healing in HIV condition, from a population-based national outlook, suggests a substantial loss of productive manpower, decline in the overall national productivity, economic development, gross domestic product, and QoL with a predictable negative impact on the poverty level. Therefore, any interventions that could prevent/ameliorate/reverse the adverse effects of HIV and HAART-related bone demineralization should be of socio-economic significance.

### 6.2 Dissemination of results

The results from this study will be communicated to health professionals via publications and presentation at conferences and seminars and to the participants via health promotion programmes.

## Acknowledgments

We would like to acknowledge the support of the Chris Hani Baragwanath Hospital, Johannesburg, South Africa and the University Teaching Hospital Enugu, Nigeria.

## Author contributions

**Conceptualization:** Adedayo Tunde Ajidahun, Hellen Myezwa, Sam Chidi Ibeneme, Sebastian Magobotha, Gerhard Fortwengel, Maxwell Jingo, Brenda Milner, Sadiya Ravat, Ifeoma Okoye, Edward Schnaid, Faith Bischoff.

**Funding acquisition:** Adedayo Tunde Ajidahun, Hellen Myezwa, Sebastian Magobotha.

**Investigation:** Sebastian Magobotha.

**Methodology:** Adedayo Tunde Ajidahun, Hellen Myezwa, Sam Chidi Ibeneme, Sebastian Magobotha, Gerhard Fortwengel, Maxwell Jingo, Brenda Milner, Sadiya Ravat, Ifeoma Okoye, Edward Schnaid, Faith Bischoff.

**Project administration:** Adedayo Tunde Ajidahun, Hellen Myezwa, Gerhard Fortwengel, Maxwell Jingo, Brenda Milner.

**Supervision:** Hellen Myezwa, Sam Chidi Ibeneme, Sebastian Magobotha, Gerhard Fortwengel.

**Writing – original draft:** Adedayo Tunde Ajidahun, Hellen Myezwa, Sam Chidi Ibeneme, Sebastian Magobotha, Gerhard Fortwengel, Faith Bischoff.

**Writing – review & editing:** Adedayo Tunde Ajidahun, Hellen Myezwa, Sam Chidi Ibeneme, Sebastian Magobotha, Gerhard Fortwengel, Maxwell Jingo, Brenda Milner, Sadiya Ravat, Ifeoma Okoye, Edward Schnaid.
